# Gaining Insights Into Metabolic Networks Using Chemometrics and Bioinformatics: Chronic Kidney Disease as a Clinical Model

**DOI:** 10.3389/fmolb.2021.682559

**Published:** 2021-05-14

**Authors:** Julien Boccard, Domitille Schvartz, Santiago Codesido, Mohamed Hanafi, Yoric Gagnebin, Belén Ponte, Fabien Jourdan, Serge Rudaz

**Affiliations:** ^1^School of Pharmaceutical Sciences, University of Geneva, Geneva, Switzerland; ^2^Institute of Pharmaceutical Sciences of Western Switzerland, University of Geneva, Geneva, Switzerland; ^3^Translational Biomarker Group, Department of Internal Medicine Specialties, University of Geneva, Geneva, Switzerland; ^4^Unité Statistique, Sensométrie et Chimiométrie, Nantes, France; ^5^Service of Nephrology and Hypertension, Department of Medicine, Geneva University Hospitals (HUG), Geneva, Switzerland; ^6^Toxalim, Research Centre in Food Toxicology, Université de Toulouse, INRAE, ENVT, INP-Purpan, UPS, Toulouse, France

**Keywords:** metabolomics, chemometrics, bioinformatics, integrative data analysis, chronic kidney disease, metabolic networks

## Abstract

Because of its ability to generate biological hypotheses, metabolomics offers an innovative and promising approach in many fields, including clinical research. However, collecting specimens in this setting can be difficult to standardize, especially when groups of patients with different degrees of disease severity are considered. In addition, despite major technological advances, it remains challenging to measure all the compounds defining the metabolic network of a biological system. In this context, the characterization of samples based on several analytical setups is now recognized as an efficient strategy to improve the coverage of metabolic complexity. For this purpose, chemometrics proposes efficient methods to reduce the dimensionality of these complex datasets spread over several matrices, allowing the integration of different sources or structures of metabolic information. Bioinformatics databases and query tools designed to describe and explore metabolic network models offer extremely useful solutions for the contextualization of potential biomarker subsets, enabling mechanistic hypotheses to be considered rather than simple associations. In this study, network principal component analysis was used to investigate samples collected from three cohorts of patients including multiple stages of chronic kidney disease. Metabolic profiles were measured using a combination of four analytical setups involving different separation modes in liquid chromatography coupled to high resolution mass spectrometry. Based on the chemometric model, specific patterns of metabolites, such as N-acetyl amino acids, could be associated with the different subgroups of patients. Further investigation of the metabolic signatures carried out using genome-scale network modeling confirmed both tryptophan metabolism and nucleotide interconversion as relevant pathways potentially associated with disease severity. Metabolic modules composed of chemically adjacent or close compounds of biological relevance were further investigated using carbon transfer reaction paths. Overall, the proposed integrative data analysis strategy allowed deeper insights into the metabolic routes associated with different groups of patients to be gained. Because of their complementary role in the knowledge discovery process, the association of chemometrics and bioinformatics in a common workflow is therefore shown as an efficient methodology to gain meaningful insights in a clinical context.

## Introduction

While efforts are still being made to improve both technological and computational aspects, metabolomics is now recognized as an essential approach to assess biochemical phenotypes in many application fields, including clinical research. Mass spectrometry (MS) has established itself as a major analytical detection technique by offering high sensitivity and substantial throughput (Zhang et al., 2020). Metabolomic experiments often generate large amounts of high-dimensional and complex biochemical data involving multiple signals measured from thousands of low molecular weight compounds. Dedicated strategies need thus to be applied to extract meaningful biological knowledge from the collected MS data (Boccard et al., 2010). Despite the considerable developments made to improve the different steps of the workflow (Pezzatti et al., 2020), assessing the metabolic diversity of a complex sample still constitutes a challenging analytical endeavor. The difficulty is mainly due to the large chemical space and concentration ranges covered by metabolites characterizing biological systems (Frainay et al., 2018). The integration of data collected from different sample preparation protocols, separation principles, ionization modes or analytical platforms has been recognized as an efficient strategy to improve the metabolome coverage of complex samples, thus potentially offering better understanding of the underlying biological mechanisms associated with a given phenotypic pattern (Richards et al., 2010). Dedicated data mining tools accounting adequately for metabolomic signals spread over multiple data tables are therefore needed, and chemometrics offers potent solutions for data integration based on dimensionality reduction approaches. More specifically, multivariate models able to handle multiple blocks of variables (multiblock) associated with different groups of observations (multigroup) are now established as effective methods for data integration in omics disciplines (Boccard and Rudaz, 2014).

An additional complexity frequently encountered in clinical research is due to a certain degree of heterogeneity in sample collection among different groups of individuals. Classical case–control studies usually involve a group of healthy volunteers used as a reference and compared to one or various pathological situations. On one hand, it could be difficult to collect measurements obtained by a highly invasive technique for the control group. On the other hand, a longitudinal follow-up of critical patients involving repeated measures at different time points may be required in standard protocols. This temporal follow-up offers rich information, but these types of longitudinal setups generate multiway data, i.e., three-dimensional tensors in this case (individuals × variables × time) making the global analysis of all available data more challenging.

In this context, Network Principal Component Analysis (NetPCA) has been recently proposed as a novel and generic approach to handle any type of structure composed of several data matrices (Codesido et al., 2020). Relationships between groups of observations or blocks of variables are translated into a network structure, where the nodes are standard two-dimensional data matrices. Two types of edges link the nodes to connect data tables characterized by the same observations or variables. This formalism translates any links between data matrices into optimization constraints to find principal directions of covariations by using the same model parameters (i.e., coefficients or loadings), that are then extracted using a set of linear models.

Handling data spread over multiple matrices constitutes a crucial step toward insightful data integration, but biological information is rarely obtained directly from statistical models, e.g., based on variables coefficients, or selected subsets of annotated metabolites. It is now well-recognized that a contextualization of metabolomic results is mandatory to go beyond simple associations and provide reliable mechanistic hypotheses (Kell, 2004). Converting subsets of up- or down-modulated biomarkers into biological processes and functions plays thus another critical role to go toward a functional description of the molecular events under study (Booth et al., 2013).

A first approach to this aim is to derive a biological meaning from a subset of relevant metabolites by retrieving metadata associated with each compound, such as chemical classes or known metabolic pathways. This can be done using a controlled vocabulary, i.e., ontologies allowing functional annotations, and bioinformatic tools that are designed to query this information stored in dedicated databases, e.g., KEGG (Kanehisa and Goto, 2000) and HMDB (Wishart et al., 2018). Biological processes and/or molecular functions can then be ranked using a statistical test (e.g., Fisher exact test or hypergeometric test) according to their probability to be represented more frequently than it would occur by random chance (Kankainen et al., 2011). The rationale behind this strategy is that the metabolites belonging to a metabolic pathway involved in the manifestation of a specific metabolic signature are expected to be modulated simultaneously. This is a rather strong hypothesis that may not be fulfilled in a real biological context and this type of analysis has therefore some limitations (Marco-Ramell et al., 2018).

Alternatively, describing metabolic networks as graphs constitutes a very efficient methodology to model biochemical reactions defining the metabolism. A metabolite-centric representation can be gained using a compound graph defining metabolites as nodes, which are connected if they are substrate and product of the same reaction (Guimera and Amaral, 2005). Metabolic pathways can then be defined as subgraphs involving a series of metabolites belonging to the same metabolic process. Such a strategy allows to go much further in the biological interpretation of altered metabolic patterns, compared to standard metabolic pathways over-representation analysis. As enzymes drive most of these reactions, they can be related to specific proteins, which are synthesized from their corresponding genes. These biological links between genes and metabolites constitute valuable information that can be used to infer metabolic networks from genomes, following a systems biology approach. Major improvements of the sequencing technology and databases have indeed enabled the reconstruction of genome-scale metabolic networks of several model organisms (Yilmaz and Walhout, 2017). It is, however, to be noted that network curation and validation remain mandatory to correct for false positive or missing reactions, and guarantee an adequate stoichiometric balance. Various metrics can then be applied to highlight specific topological features of interest, such as path lengths, degree centrality and clustering coefficient, which allow hubs and modules of topological importance to be highlighted (Lacroix et al., 2008). Moreover, subnetworks focusing on specific sets of reactions related to modulated metabolites can be easily extracted and visualized (Frainay and Jourdan, 2017). Such a strategy is useful to reduce the complexity of large networks and to gain a better mechanistic understanding of the phenomenon under study, through the possibility to investigate a reduced list of biochemical reactions characterizing particular metabolic phenotypes. Resources for pathway mapping and/or network analysis include KEGG (Kanehisa and Goto, 2000), MetaCyc (Caspi et al., 2016), Recon (Thiele et al., 2013; Noronha et al., 2017), and MetExplore (Cottret et al., 2010, 2018).

This work presents the integrative analysis of data collected from several metabolomic studies designed to investigate the effects of chronic kidney disease (CKD). The selected analytical strategy was based on multiple chromatographic separation setups, including reversed-phase liquid chromatography (RPLC), hydrophilic interaction chromatography with amide (aHILIC), and polymeric zwitterionic stationary phases (ZICpHILIC) coupled to high-resolution mass spectrometry (HRMS). The heterogeneous and complex data structure generated was successfully handled using chemometrics for multiblock and multiset data integration, and state-of-the-art bioinformatic resources were implemented for an in-depth study of metabolic events.

## Materials and Methods

### Chronic Kidney Disease Dataset

Plasma samples collected during four prospective observational monocentric CKD studies performed at a tertiary hospital (Geneva University Hospitals, Geneva, Switzerland) were considered. CKD severity stages were defined according to the glomerular filtration rate (GFR) criterion KDIGO guidelines (Levey et al., 2020). A first cohort of patients was recruited to assess the impact of different stages of CKD severity (3b–5) on plasma metabolites (Gagnebin et al., 2019). A second and third one explored the benefits offered by kidney transplantation, as well as the potential impact of kidney donation on healthy living donors (LKD) (Gagnebin et al., 2020b). Finally, the last one was designed to investigate plasmatic metabolites patterns of patients with end-stage renal disease (ESRD) undergoing regular hemodialysis (HD) (Gagnebin et al., 2020a). The latter were under chronic HD for at least 3 months with three dialysis per week. In addition, a control group of healthy volunteers was also included.

Plasma from all cohorts were collected for an integrative analysis of these different renal conditions. All samples were collected in the morning after an overnight fast and several times points were considered for individuals undergoing HD or transplantation, as well as LKD. Samples were directly thawed, aliquoted and stored at –80°C. More details about the inclusion criteria, GFR, HD characteristics and ethical concerns can be found in the specific reference of each study.

### Sample Preparation and Analysis

#### Sample Preparation

Pipetting and liquid handling was carried out using a Tecan Freedom Evo-2 (Tecan, Switzerland) to ensure sample preparation repeatability. First, solvent protein precipitation was performed using cold methanol spiked with isotopically labeled standards [(d_5_-indole)-L-tryptophan, 1,2-^13^C_2_-taurine and 2,2,4-d_3_-DL-glutamic acid)], all from Cambridge Isotope Laboratories Inc. (Andover, United States) at 1.25 μg mL-1. A volume of 960 μL cold methanol containing the standards was added to 240 μL of thawed plasma. Samples were then vortexed for 20 s, mixed at 1,200 rpm for 30 min at 4°C and centrifuged at 15,000 × g for 20 min at 4°C. Supernatants were divided into 300 μL aliquots and dispatched on 96-well plates. Each extract was then dried for 8 h in a Thermo Fisher Scientific Savant 210A SpeedVac (Thermo Electron LED GmbH, Langenselbold, Germany) and stored at –80°C. Before analysis, samples were reconstituted in 60 μL of H_2_O/MeCN (95:5, v/v) for RPLC and in 60 μL of H_2_O:MeCN (25:75, v/v) for aHILIC and ZICpHILIC. Quality control (QC) and diluted QC (dQC) samples were prepared to assess and correct analytical variations using the same sample preparation procedures as the individual samples.

#### LC-MS Analysis

Liquid chromatography was carried out on a Waters H-Class Acquity UPLC system (Waters Corporation, Milford, MA, United States) using different separation modes. RPLC analysis was performed using a Phenomenex Kinetex C18 column (150 × 2.1 mm, 1.7 μm), and a SecurityGuard ULTRA pre-column. A gradient of mobile phase A (0.1% FA in water) and mobile phase B (0.1% FA in MeCN) was applied as follows: 2% B for 1 min, increased to 100% B over 14 min, held for 3 min, and then returned to 2% B to re-equilibrate the column for 7 min (total run time of 25 min) at a flow rate of 300 μL min-1 and a column temperature of 30°C. Separation using aHILIC was achieved on a Waters Acquity BEH Amide column (150 × 2.1 mm, 1.7 μm), and a VanGuard^TM^ pre-column. A gradient of mobile phase A (H_2_O:MeCN; 5:95 v/v) and mobile phase B (10 mM ammonium formate in H_2_O:MeCN, 70:30 v/v adjusted at pH 6.50 in the aqueous part) was applied as follows: 0% B for 2 min, increased to 70% B over 18 min, held for 3 min, and then returned to 0% B to re-equilibrate the column for 7 min (total run time of 31 min) at a flow rate of 500 μL min-1 and a column temperature of 40°C.

ZICpHILIC analysis was carried out on a Merck SeQuant Zic-pHILIC column (150 × 2.1 mm, 5 μm) and the appropriate guard kit was applied. A gradient of mobile phase A (MeCN) and mobile phase B (2.8 mM ammonium formate adjusted to pH 9.00) was applied as follows: 5% B for 1 min, increased to 51% B over 9 min, held for 3 min at 51% B and then returned to 5% B in 0.1 min before re-equilibrating the column for 6.9 min (total run time of 20 min) at a flow rate of 300 μL min-1 and a column temperature of 40°C. For all separation modes, a volume of 5 μL of the 393 samples was analyzed in eight batches using constrained randomization (Jonsson et al., 2015). QC samples were injected for system conditioning (15 first injections in each batch), while QCs and dQCs were analyzed regularly during the sequence (every 6 samples).

The UPLC system was coupled to a maXis 3G Q-TOF high-resolution MS from Bruker (Bruker Daltonik GmbH, Bremen, Germany) with an electrospray ionization source working in positive (ESI+) mode for RPLC and aHILIC, or negative (ESI−) mode for RPLC and ZICpHILIC. The instrument was operated using the following parameters: capillary voltage of –4.7 kV for ESI+ and 2.8 kV for ESI-, nebulizing gas pressure of 2.0 bar, drying gas temperature of 225°C for RPLC and 200°C for aHILIC and ZICpHILIC and a flow rate of 8.0 L min^–1^. Data acquisition between 50 and 1,000 m/z was performed in profile mode at a rate of 2 Hz. In-run automatic calibration was achieved using formate adducts in the 90–1,247 m/z range and the quadratic plus high-precision calibration algorithm (Bruker Daltonics). The detailed analytical protocol can be found elsewhere (Gagnebin et al., 2019).

#### Data Processing and Analysis

Raw data processing was performed using Progenesis QI 2.3 (Non-linear Dynamics, Waters, Newcastle upon Tyne, United Kingdom). QCs and dQCs were used to monitor and control data acquisition quality, remove unreliable signals, and correct for within-batch drifts and between-batch effects. A filtering procedure was carried out to remove unreliable signals by applying a threshold of 50% for the dQC/QC ratio relative standard deviation (RSD) and a dQC/QC ratio between 0.2 and 0.8. LOESS regression was used for intra- and inter-batch normalization based on QC samples. Finally, the position of the QCs was assessed separately for each data block using Principal Component Analysis (data not shown).

Metabolite annotation was achieved using an in-house database containing experimental data from more than 900 authentic standard compounds measured in different chromatographic conditions. Briefly, level 1 annotation was achieved by matching *m/z* values, retention times, and isotopic patterns, while MS/MS spectra and collisional cross-section values were considered for confirmatory purpose. Cytoscape 3.8.2 was used to generate circular layout graphs. NetPCA was computed after unit variance scaling using the NetPCA Python package (Codesido et al., 2020).

Clustering was computed under the MATLAB 9.5 environment (The MathWorks, Natick, United States). MetExplore (Cottret et al., 2010) and MetExploreViz (Chazalviel et al., 2018) were used with the Recon3D human metabolic reconstruction (Brunk et al., 2018) for mapping identified metabolites, pathway over-representation analysis, network visualization and evaluation of carbon transfer reaction paths. A more detailed description of the data processing and analysis workflow, as well as the dataset, are available as Supplementary Material.

## Results

### Metabolomic Dataset Structure

In total, 393 blood samples were collected from the pre-defined cohorts of patients grouped into different categories according to their renal status: 56 healthy control volunteers (CTRL), 69 CKD patients at intermediate stage (ICKD), 35 patients with ESRD undergoing HD (HD), 42 ESRD undergoing kidney graft (KG) and 24 healthy LKD (DV). Repeated measures were carried out for three groups: (i) HD: before (preHD) and after (postHD) blood dialysis on the mid-week session, (ii) KG: before (preKG), 1 week (postKG1), and 1 month (postKG2) after the graft, (iii) DV: before (preDV), 1 week (postDV1) and 1 year (postDV2) after the transplantation procedure.

**FIGURE 1:**
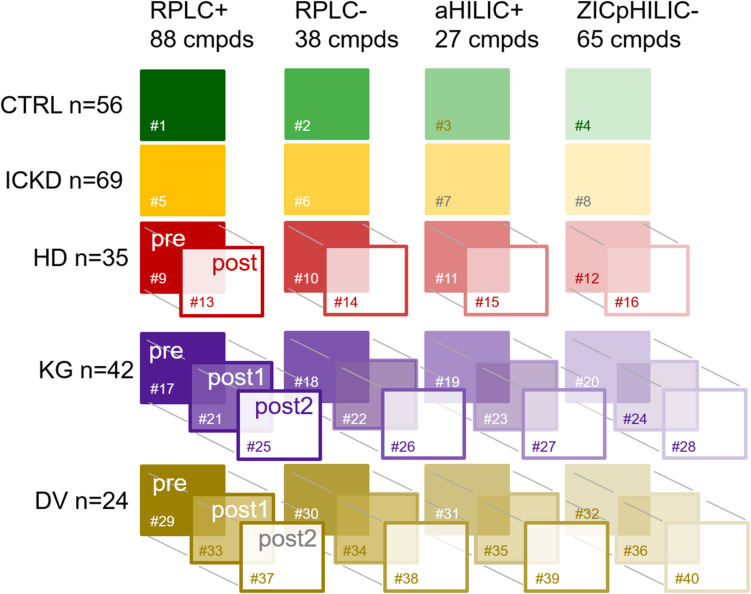
Schematic diagram of the dataset structure composed of 40 data matrices involving the five different groups of individuals, the four blocks of variables, and repeated measurements. CTRL, control group; ICKD, intermediate chronic kidney disease; HD, hemodialysis; KG, kidney graft; DV, living kidney donors. Sharps indicate the numbering of the tables.

All samples went through our standard analytical workflow already presented elsewhere (Pezzatti et al., 2020). Briefly, generic sample preparation was first carried out to cover a large chemical diversity of compounds and each sample was analyzed using four LC-HRMS setups, i.e., RPLC+, RPLC−, aHILIC+, and ZICpHILIC-. Raw data processing involving baseline correction, peak picking, adduct deconvolution and retention time alignment was carried out with Progenesis QI, while subsequent filtering and normalization based on QC samples (Broadhurst et al., 2018; Pezzatti et al., 2020) was performed using in house scripts. Metabolite annotation was then performed by matching reference values measured with standards in the same analytical conditions, and a scoring strategy was implemented to remove compounds annotated in more than one LC-MS setup. For that purpose, a peak quality score based on intensity, shape, and retention time was used to select the best analytical information (Pezzatti et al., 2019). In the present work, annotation was restricted to metabolites matching entries from our in-house database. This strategy led to a set of 218 compounds annotated at level 1 for each sample. These identified metabolites were spread over four blocks of variables corresponding to the different LC-MS setups as follows: 88 in RPLC+, 38 in RPLC−, 27 in aHILIC+, and 65 in ZICpHILIC-. A schematic diagram of the dataset structure composed of 40 data matrices involving the five different groups of individuals, the four blocks of variables, and repeated measurements for HD, KT, and KD is provided in Figure 1.

### Network Principal Component Analysis Modeling

**FIGURE 2:**
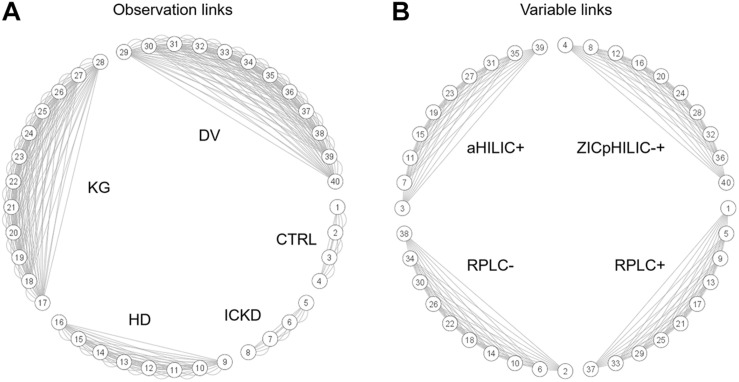
Circular layout graphs describing network connections between **(A)** groups of samples and **(B)** blocks of variables. The numbering of the data matrices corresponds to that given in Figure 1.

This type of complex data structure involving multiple groups of observations and blocks of variables, together with repeated measures for certain individuals, is not straightforward to handle efficiently, i.e., without breaking connections between data matrices linking their rows and/or columns. NetPCA was performed after unit variance scaling using connection links between data matrices to define the constraints of the model. For that purpose, a network incidence matrix was used to define the topology of the data structure. By these means, matchings between the same sets of observations (rows), variables (columns), or both (rows and columns) could be explicitly included in the optimization process, thus leading to global components accounting for these links. Circular layout graphs corresponding to the connections between (A) groups of observations and (B) blocks of variables are presented in Figure 2. Using this representation, blocks of data associated with the same individuals appear clearly (data matrices #1–4 for CTRL and #5–8 for ICKD), while tensorial substructures generated by repeated measures during the longitudinal follow-ups form larger clusters (data matrices #9–16 for HD, #17–28 for KG, and #29–40 for DV). Moreover, the four subsets of metabolites measured using the different LC-MS setups were also clearly visible.

**FIGURE 3:**
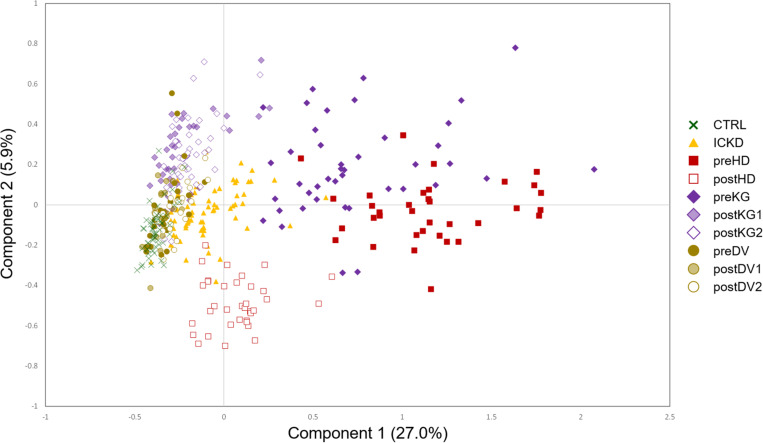
NetPCA score plot of the two first global components. CTRL, dark green crosses; ICKD, filled orange triangles; preHD, filled red squares; postHD, empty red squares; preKG, filled violet diamonds; postKG1, light violet diamonds; postKG2, empty violet diamonds; preDV, filled brown circles; postDV1, light brown circles; postDV2, empty brown circles.

A two-component NetPCA model was considered to investigate the main metabolic variations in the dataset. From the distribution of the groups on the score plot (Figure 3), it appeared that the first component, summarizing 27.0% of the total variability, strongly followed the severity of CKD. The CTRL and preDV groups of healthy individuals were located on the left (negative scores), ICKD transitional situation in the middle, while preHD and preKG patients characterized by ESRD requiring specific treatment were distributed on the right (positive scores). This important part of explained variance is consistent with prior knowledge regarding the massive kidney dysfunction associated with CKD status characterized by markedly decreased glomerular filtration. These characteristics are indeed shared by the preKG and preHD groups with the most severe kidney damage. It should also be noted that postHD samples are associated with a return to a situation similar to ICKD along this first component, compared to the preHD group. A similar observation can be made by comparing the samples of the KG group before (preKG) and after (postKG1 and postKG2) transplantation. As patients seem to recover (at least partially) a metabolic profile similar to less severe stages of renal disease, this suggests marked beneficial effects offered by hemodialysis and graft on many metabolite levels. In addition, kidney donation does not appear to affect the metabolomic profile of LKD over the long term, as postDV1 and postDV2 groups of samples were located close to healthy individuals on the left, with only slightly higher metabolites levels possibly resulting from reduced filtration capacity with a single kidney.

Finally, samples from the postHD group, i.e., metabolic profiles measured after dialytic therapy, are characterized by negative scores on the second component on which they appear separated from the other sample groups, revealing thus another pattern of metabolic profiles that was not observed in the case of endogenous renal alteration. It is to be noted that the second component summarized a trend driven only by a subset of samples with 5.9% of the total variability, thus more specific to the impact of hemodialysis. The NetPCA score plot of the two first global components is depicted in Figure 3.

Relative block influences were computed for both components, thus offering an objective way to evaluate the relative contributions of each data matrix to the global decomposition. Prior observations were confirmed, as the first component was mainly associated with variations from data blocks related to the preHD and preKG groups, while the second component summarized the trend associated with postHD samples. Interestingly, balanced contributions from the four LC-MS setups could be highlighted, underlining their overall agreement in terms of biochemical information. Relative blocks influences are summarized in Table 1.

**TABLE 1:** Relative block influences for the two first NetPCA components.

	NetPCA Component 1				NetPCA Component 2			
Group	RPLC+	RPLC−	aHILIC+	ZICpHILIC−	RPLC+	RPLC−	aHILIC+	ZICpHILIC−
CTRL	2.6%	2.6%	2.4%	2.3%	1.4%	1.5%	1.0%	1.6%
ICKD	0.3%	0.3%	0.3%	0.1%	0.1%	0.1%	0.1%	0.3%
preHD	13.3%	13.7%	13.1%	15.1%	0.1%	0.1%	0.0%	0.0%
postHD	0.0%	0.1%	0.1%	0.1%	11.2%	12.0%	14.0%	15.7%
preKG	4.6%	6.4%	2.2%	7.6%	2.3%	1.7%	0.7%	3.3%
postKG1	0.8%	0.8%	0.7%	0.4%	3.8%	4.1%	4.2%	4.0%
postKG2	0.5%	0.5%	0.4%	0.4%	3.8%	3.6%	3.5%	4.7%
preDV	0.7%	0.8%	0.7%	0.6%	0.1%	0.1%	0.1%	0.0%
postDV1	0.9%	0.9%	0.9%	0.8%	0.2%	0.2%	0.2%	0.1%

### Metabolite-Centric Analysis

The contributions of the variables to the components (or loadings) are helpful to investigate the trends associated with meaningful observations groupings and interpret multivariate models. Because NetPCA accounts explicitly for multigroup structures, these influences of the variables can be computed by taking only specific subsets of observations into account, i.e., subsets of metabolites associated with a specific renal status in this case. This provides also an objective basis to highlight specific differences between two groups, e.g., CTRL and postHD, by assessing potentially dissimilar patterns of compounds summarizing the variability of the two metabolic phenotypes. Based on this information, it is also possible to adopt another way of interpreting the model through a variable-centric approach. In this case, the contribution of each metabolite to the explained variability can be summarized using the different components of the model. A cumulative contribution for each component, according to its relative part of variability explained can be expressed as a percentage of the total variance of the metabolite. Ranking the variables according to their percentage of variance explained for a given component of interest allows a straightforward extraction of the biological variability of interest. Explicitly accounting for the data structure provides thus a better understanding of the role of each variable in the decomposition. The percentage of explained variance for the 218 metabolites is summarized in Supplementary Table 1. A threshold of 20% was applied in order to underline relevant metabolites accounting for at least this proportion of their total variability on the different axes. This led to a large subset of 106 compounds markedly modulated according to disease severity on the first component, while a smaller pattern composed of 14 metabolites was associated with the second axis. From these results, it can be observed that a massive increase of the abundance of a large number of metabolites is associated with first component. This general accumulation of blood metabolites is in line with prior knowledge of the pathology (Zhao, 2013).

Levels of known uremic retention solutes, such as creatinine, adipate, allantoin, or hippuric acid were observed as positively correlated with the first component, i.e., levels that were increased according to disease severity (Boelaert et al., 2013). Moreover, numerous N-acetyl amino acids were also in the subset of metabolites with markedly augmented abundances, including N-acetylmethionine, N-acetyltryptophan, N-acetylphenylalanine, N-acetylleucine, N-acetylproline, N-acetyllysine, N-asparagine. These may be the hallmark of N-acetylation as an altered detoxification mechanism in CKD (Sekula et al., 2016). Other noticeably increased metabolites were kynurenic acid, formylmethionine, anthranilic acid, 5’-methylthioadenosine, myo-inositol and indoxyl sulfate. Tryptophan and guanidinoacetic acid (a precursor of creatine, creatinine and urea) were characterized by a decrease level when compared to healthy volunteers with fully efficient kidney function. Overall, these results were in agreement with previous studies (Hocher and Adamski, 2017; Kalim and Rhee, 2017).

Additionally, a subset of metabolites was highlighted as characteristic of the dialyzed patients (postHD), on the second component, involving various amino acids, such as lysine, arginine, methionine, proline, threonine, cysteine, valine and norvaline. Interpretation remains, however, challenging, as this observation could result from lower overall metabolite concentration and reduced ion suppression that may increase signal (Gagnebin et al., 2017). Moreover, HD patients were under chronic hemodialysis for at least 3 months with standard three dialysis per week, and this effect could be due to a cyclic equilibria of metabolite levels, as discussed with the medical team. An evaluation of the net balance would require quantitative plasmatic measurements as well as proper assessment of clearance.

### Bioinformatics

In order to go beyond simple lists of potential biomarkers and propose biological hypotheses, further analysis of metabolic pathways was carried out using MetExplore (Cottret et al., 2010). The latter uses biosources, i.e., curated metabolic networks obtained from genome-scale reconstructions, to offer deeper insights into metabolic modulations and a better understanding of potential mechanisms leading to a specific metabolic phenotype. The Recon3D (Brunk et al., 2018) biosource of *Homo sapiens* derived from 2,990 genes was used as the most accurate reconstruction of the human metabolic network to date. Recon3D topology includes the cellular localization of metabolites (e.g., mitochondria, cytoplasm, etc.) but this description of biological compartments is not relevant for biological matrices such as plasma. A simplified version of the network was obtained by removing this information, and a single node was considered for each metabolite in the case of multiple occurrences in different compartments. This network was composed of 109 pathways involving 4,095 metabolites, 5,389 reactions and 3,099 enzymatic complexes. A subset of 134 metabolites were successfully mapped on Recon3D using MetExplore *Metabolite Identifier Matcher* module, corresponding to 61% of the pool of compounds identified in the samples.

#### Over-Representation Analysis

Potential biomarker metabolites related to the first components were investigated using over-representation analysis. The latter aims to highlight pathways the more likely associated with a subset of metabolites by assessing whether they contain significantly more differentially expressed compounds than expected by chance. Because hemodialysis cannot be considered as a biological process, differences between preHD and postHD conditions summarized by the second component should indeed not be associated with specific biological pathways. Therefore, it was not relevant to further investigate potentially impacted metabolic pathways.

**FIGURE 4:**
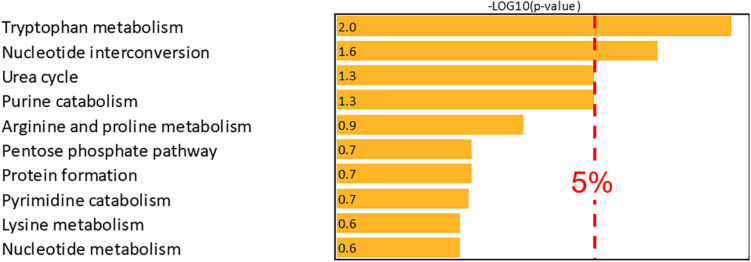
Over-representation analysis (–log10 of the right-tailed Fisher test *p*-value corrected using the Benjamini-Hochberg False Discovery Rate procedure). The –log10 value of the 5% significance threshold is 1.301.

Metabolite mapping allowed 55 compounds associated with the first NetPCA component to be localized on the Recon3D network and over-representation analysis was therefore performed based on this subset. By these means, two pathways were reported as over-represented, namely *tryptophan metabolism* and *nucleotide interconversion*. The latter were thus objectively confirmed as metabolic pathways associated with CKD with 7 and 6 metabolites present in the subset of potential biomarkers, respectively. Moreover, *urea cycle* and *purine catabolism* were close to the significance threshold. A summary of the results of the over-representation analysis is proposed in Figure 4.

#### Network Analysis

Investigating metabolic pathways characterizing specific phenotypes provide valuable information about biological processes but are, however, limited to offer a global overview of the metabolism and its potential alterations. As many metabolites are involved in multiple interconnected pathways, the specificity of a metabolic signature is often difficult to guarantee. This is particularly crucial in a case such as CKD, because relevant phenotypic features of the disease may be spread over several pathways. As a consequence, it could be challenging to gain an overall understanding of the molecular events based only on pathway analysis.

Metabolic networks connecting all the pathways as a single object constitute therefore a very interesting alternative strategy. Subnetwork extraction was carried out based on significantly over-represented metabolic pathways associated with the first NetPCA component, namely *tryptophan metabolism* and *nucleotide interconversion*. By these means, it was then possible to highlight hubs, modules and bridges from the topology of the graph. This was done by investigating paths between pairs of metabolites, as a sequence of edges (i.e., reactions) connecting the starting node to the ending node. Due to the high number of edges composing a metabolic network, a very large amount of paths is possible, but by far not all of them are biologically relevant. Following the parsimony principle, the shortest path between two compounds might seem to be a suitable answer to make a choice among this multiplicity of alternatives. However, in many situations this solution is biochemically not optimal. Therefore, path search algorithms have been developed by incorporating biochemical rules to find the most relevant metabolic routes. Investigating lightest paths constitutes an efficient strategy, and it was performed based on the evaluation of the minimal squared degree sum of the nodes in the path, thus avoiding to give too much emphasis to uninformative ubiquitous compounds (Croes et al., 2006).

**FIGURE 5:**
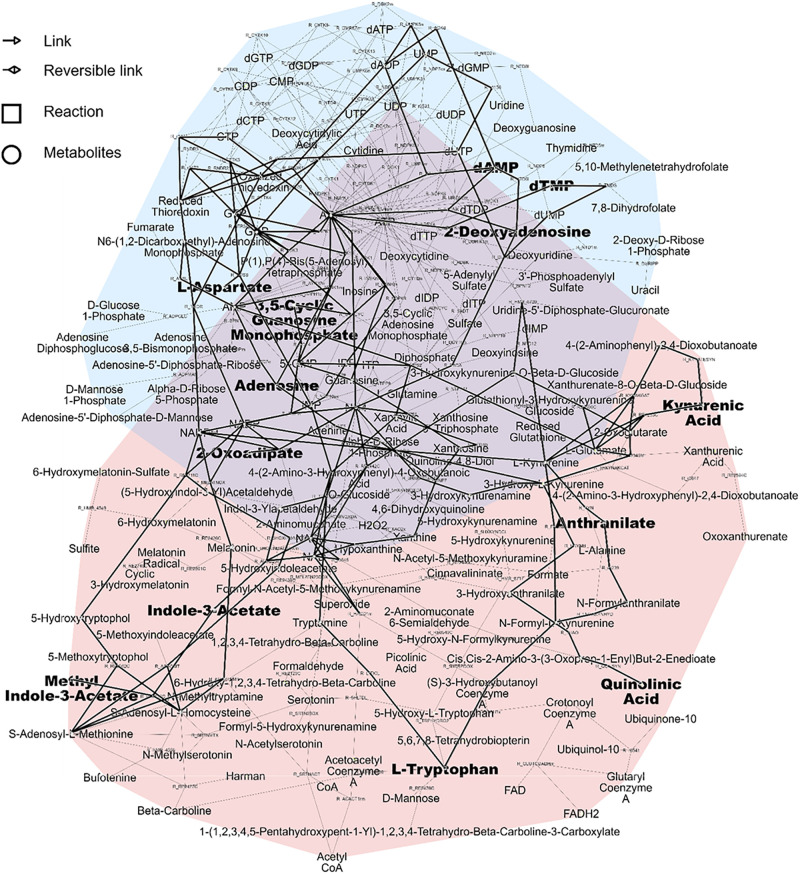
Pink pathway: Tryptophan metabolism. Blue pathway: Nucleotide interconversion (purine and pyrimidine metabolism). Metabolites related to the first NetPCA component and the corresponding subnetwork are shown in bold.

As a result, the two pathways highlighted as the most relevant using over-representation analysis, namely *tryptophan metabolism* and *nucleotide interconversion* could be efficiently displayed and linked in the subnetwork related to the first NetPCA component. Interestingly, the central role played by nucleotides and their derivatives could be highlighted, as metabolic hubs involved in a large number of reactions. Moreover, the reaction paths between tryptophan, kynurenic acid and indole-3-acetate are in line with prior studies reporting the link between renal dysfunction and the enzymatic activity of indoleamine 2,3-dioxygenase. The latter is responsible for tryptophan catabolism, the initial molecular event of the kynurenin pathway (Schefold et al., 2009). A decreased tryptophan plasmatic level is associated with CKD, while increased abundances of downstream products such as kynurenic acid have been reported (Kalim and Rhee, 2017). Related to these findings, a protein-bound uremic toxin from gut microbial origin, namely indoxyl sulfate was also highlighted as a metabolite of tryptophan associated with kidney failure (Niwa et al., 1994). Alterations in nucleotide interconversion may involve disturbed purine and pyrimidine metabolism. These have already been linked to both an increase in the prevalence and progression of the disease (Sekula et al., 2016; Shen et al., 2016). The degradation of purine derivatives generates hypoxanthine, which is further converted to xanthine and finally to uric acid, which is in accordance with known associations with CKD and uremic solutes. The subnetwork associated with these two pathways and the subset of altered metabolites related to the first NetPCA component is presented in Figure 5.

#### Carbon Transfer Reaction Paths

**FIGURE 6:**
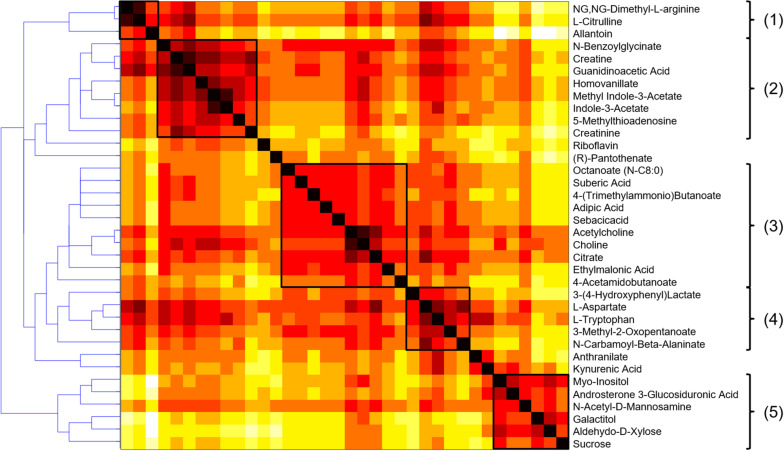
HCA dendrogram and heatmap of the metabolic fingerprint distance matrix, with the five clusters highlighted as relevant biological modules. The darker, the shorter reaction path.

Investigating the importance of individual nodes in a metabolic network is often challenging because these parameters are correlated with high degrees, therefore giving preference to highly connected pathways in terms of global topological properties. A node having many connections may therefore lack specificity in terms of metabolic information and not be a key determinant for a given process. Ubiquitous compounds playing an auxiliary role in metabolic reactions, e.g., H_2_O, CO_2_, or NAD, often constitute shortcuts in the graph and this also includes compounds associated with the *nucleotide interconversion* pathway, i.e., AMP, ADP, and ATP, as well as their deoxy counterparts. As it makes the investigation of local connectivity and bridges between modules challenging, a list of such side compounds was excluded from further topological investigations. Based on this, atom mapping was used for each reaction to evaluate the transfer between substrate and product atoms (Rahman et al., 2016), and edges not supporting any carbon atom transfer were removed from the compound graph. Conversely, those meeting this criterion were further considered as relevant for topological analysis (Frainay and Jourdan, 2017). Metabolic modules were then evaluated based on their reaction path in this graph using a distance matrix computed to summarize the different reaction paths between the compounds of the selected metabolic subset. Hierarchical clustering was then carried out on the distance matrix using complete-linkage to highlight potential biologically meaningful groupings. This agglomeration strategy helps to find compact clusters and avoid the chaining effect that would make the detection of biological modules more difficult. The HCA dendrogram and heatmap are presented in Figure 6.

A visual inspection of the dendrogram revealed five main clusters: (1) a small cluster composed of 3 amino acids of the urea cycle (N.N-dimethyl-L-arginine, L-citrulline and allantoin), that was connected with (2) a group of 8 metabolites associated with creatine metabolism (creatine, creatinine, guanidoacetic acid, hippuric acid, homovanillic acid, methyl indole-3-acetic acid, indole-3-acetic acid, 5-methylthioadenosine). It is well-known that creatinine blood levels reflect glomerular filtration efficiency and the urea to creatinine ratio can be used to characterize kidney function impairment (Duarte and Preuss, 1993). It constitutes therefore a clear positive control biomarker, reinforcing the biological validity of the findings.

**FIGURE 7:**
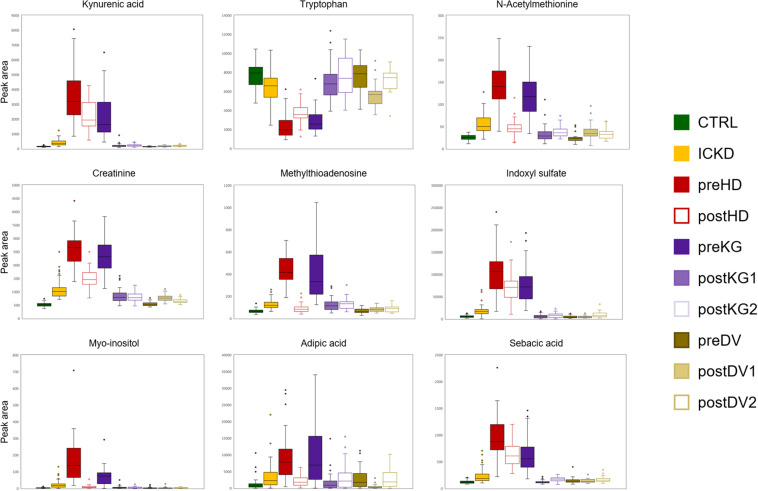
Box-plots showing nine metabolites with representative alteration patterns from altered pathways.

A third cluster (3) composed of 10 metabolites included small carboxylic acids that could be potentially linked to disorders of fatty acid oxidation (adipic acid, sebacic acid, suberic acid, caprylic acid, deoxycarnitine, acetylcholine, choline, citric acid, ethylmalonic acid, 4-acetamidobutanoic acid) (Kang et al., 2015). Moreover, carnitine is necessary to transfer fatty acids for their oxidation in the mitochondria, and it also plays an important role in acetylcholine metabolism (Hoppel, 2003). The fourth (4) subset of 5 metabolites involving L-tryptophan, 3-(4-hydroxyphenyl)lactic acid, N-carbamoyl-beta-alanine, L-aspartate, and 3-methyl-2-oxovaleric acid, could be linked to aromatic amino acids metabolism and related compounds. Finally, (5) a cluster of sugar derivatives formed the last group (myo-inositol, N-acetyl-D-mannosamine, galactitol, D-xylose, and sucrose). Sugars are associated with the pathogenesis of diabetic nephropathy, but the underlying mechanisms involved are still unclear. Detrimental effects of advanced glycation end products constitute an interesting hypothesis (Dronavalli et al., 2008), but it remains, however, to be explored to better understand the progression of CKD. Box-plots of metabolites with representative alteration patterns from altered pathways are presented in Figure 7.

## Discussion

The association of chemometrics and bioinformatics in a common workflow was shown to be an effective approach for the integrative analysis of samples collected from several groups of patients suffering from multiple stages of CKD and/or undergoing different treatments. Despite heterogeneous sample collection, NetPCA allowed all groups of individuals to be included in a global model for an overall evaluation of their metabolic phenotypes. This integrative strategy underlined similar subsets of compounds describing the beneficial metabolic effects provided by hemodialysis and kidney graft, but also, for the first time, to compare the different alteration patterns on a common scale. The latter could be related to pathological modifications due to CKD and showed that kidney donors were only moderately affected by the decline in kidney function following organ donation. Notably, N-acetylation detoxification was reported to be altered in CKD, while tryptophan metabolism and nucleotide interconversion were highlighted using over-representation analysis. Further investigation using network reconstruction allowed deeper insights into their link to be gained. Tryptophan metabolism was already reported as an altered metabolic route in CKD, with decreased tryptophan plasmatic concentration and increased levels of downstream products such as kynurenic acid. Nucleotide interconversion can be considered as a generic biological process covering both purine and pyrimidine metabolism, both known to be affected during CKD. Further information about meaningful metabolic modules was finally obtained using hierarchical clustering based on reliable reaction paths. By these means, additional hypotheses involving creatine metabolism and urea cycle, carnitine and disorders of fatty acid oxidation, as well as aromatic amino acids metabolism and sugar derivatives could be drawn.

Although the coverage of some parts of metabolic networks or chemical families still needs to be improved and/or refined (e.g., lipids), the current databases already allow a fine assessment of the interconnectivity of the different metabolic pathways and their topology. This allows to put into perspective the differences observed between samples characterizing specific clinical or experimental situations, and to go further in the biological interpretation of the regulatory networks governing phenomena of interest. In addition, the annotation of metabolic networks is being actively carried out thus offering continuous improvements to grasp the complexity of the metabolism. With this aim in sight, the association of chemometrics and bioinformatics in a common workflow will certainly play a central role in the future of metabolomics.

## Data Availability Statement

The data supporting the conclusions of this article will be made available by the authors, without undue reservation.

## Ethics Statement

The studies involving human participants were reviewed and approved by the local ethics committee (Commission cantonale d’éthique de la recherche (CCER), Geneva, Switzerland) and performed according to the Declaration of Helsinki. The patients/participants provided their written informed consent to participate in this study.

## Author Contributions

BP coordinated CKD clinical studies and sample collection. YG carried out sample preparation, LC-MS experiments. YG, SC, and JB managed data processing. SC, JB, and MH participated in chemometrics data analysis. JB, DS, and FJ participated in bioinformatics data analysis. BP, FJ, and SR managed the project. All authors participated in manuscript writing and correction.

## Conflict of Interest

The authors declare that this study received indirect partial funding from AstraZeneca. The funder was not involved in the study design, collection, analysis, interpretation of data, the writing of this article or the decision to submit it for publication.
