# Supplementary material for: Gaining Insights Into Metabolic Networks Using Chemometrics and Bioinformatics: Chronic Kidney Disease as a Clinical Model
Source: Front Mol Biosci. 2021 May 14;8:682559. doi: 10.3389/fmolb.2021.682559 (PMC8163225; doi:10.3389/fmolb.2021.682559)
Supplement: Supplementary file 3 [file Table_3.DOCX]

Gaining insights into metabolic networks using chemometrics and bioinformatics: chronic kidney disease as a clinical model

Julien Boccard, Domitille Schvartz, Santiago Codesido, Mohamed Hanafi, Yoric Gagnebin, Belén Ponte, Fabien Jourdan, Serge Rudaz

Supplementary Material: Data Processing And Analysis Workflow

**Raw data processing**

The four cohorts represented more than 500 plasma samples analyzed using 4 LC-MS protocols, leading to more than 2’000 data files of about 1 Gb each. Raw data processing involving baseline correction, peak picking, adduct deconvolution and retention time alignment was performed with Progenesis QI 2.3 (Nonlinear Dynamics, Waters, Newcastle upon Tyne, UK). Four batches of samples were analyzed in each LC-MS setup, generating tens of thousands of features for each dataset. As a first step of data cleaning, features were selected based on signal variability and response to dilution, to remove irrelevant ions, such as noisy or saturated signals.

**Data Quality Control**

Quality Control (QC) samples and diluted QCs (dQCs) were used to monitor and control data acquisition quality, remove unreliable signals, and correct for within-batch drifts and between-batch effects. For this purpose, QCs and dQCs successively injected in pairs were used to estimate signal stability and response to dilution based on dQC/QC area ratios. A filtering procedure was implemented to remove unreliable signals that did not meet a criterion of 50% for the maximum value of dQC/QC ratio relative standard deviation (RSD) and a dQC/QC ratio between 0.2 and 0.8. While dQC/QC ratios were maintained at a value of approximately one in every dilution for some features, a second group followed a nearly linear response to sample dilution, and the latter was selected for further processing.

QCs were then used to detect and correct potential within-batch analytical drifts and between-batch effects. For that purpose, LOESS regression fitted to the QCs was used in both cases. The span value was optimized automatically using cross validation and a linear fit was used. Relatively small intra-batch analytical drift and inter-batch variability was observed, highlighting the suitability and homogeneity of the chosen analytical conditions. Finally, the position of the QCs was assessed separately for each dataset using Principal Component Analysis. By these means, the analytical variability was shown as inferior to the biological variability.

**Metabolite Annotation**

Metabolite annotation was achieved using an in-house database containing experimental data from more than 900 authentic standard compounds measured in various chromatographic conditions. Briefly, level 1 annotation was achieved by matching *m/z* values, retention times, and isotopic patterns. The following tolerance parameters were applied for feature annotation using reference standards analyzed using the same experimental conditions: 5 ppm for mass matching; 0.2 min and 5% for retention time matching. Despite the orthogonality of the four chosen separation modes, several signals were present in more than one of the datasets. In this case, an investigation was performed to select the greatest signal quality with respect to intensity, retention time and peak shape. For that purpose, a peak quality score published in a prior work was used to select the optimal analytical information (Pezzatti, Gonzalez-Ruiz et al. 2019). This procedure led to a dataset of 218 univocally identified metabolites. Additional analysis of QC samples was performed on the same UHPLC instrument coupled to a TWIMS-QTOF (Vion, Waters, Manchester, UK) equipped with an ESI source to confirm the metabolites identity based on their collisional cross-section (CCS) values and MS/MS spectra as complementary information. Acquisition was performed in high definition MS^E^ mode using ion mobility.

**NetPCA**

NetPCA was computed after unit variance scaling using the NetPCA Python package (Codesido, Hanafi et al. 2020). The NetPCA method aims to model a network of data matrices by a set of linear models whose principal directions of covariations are shared. All types of relations defining shared coefficients between data tables can be implemented. The key point of NetPCA is that the sharing of the coefficients between data tables with the same groups of samples and/or blocks of variables is imposed by the fact that they are the same mathematical objects in the optimization procedure. Forty data tables (see article Figure 1) were connected to build a network according to their links in the observations/variables modes (see article Figure 2).

**Metabolic Network Analysis**

MetExplore (Cottret, Wildridge et al. 2010) and MetExploreViz (Chazalviel, Frainay et al. 2018) were used with the Recon3D human metabolic reconstruction (Brunk, Sahoo et al. 2018) for mapping identified metabolites, pathway over-representation analysis, network visualization and evaluation of carbon transfer reaction paths. The flat version, *i.e.* without compartment information, of the Recon3D network derived from 2’990 human genes was used as the most accurate reconstruction of the human metabolic network (biosource #3223). This network includes 5’389 biochemical reactions involving 4’095 metabolites and 3’099 enzyme complexes participating to 109 pathways. The *Metabolite Identifier Matcher* module was used to retrieve the metabolic network identifiers associated with the metabolites in the dataset. This tool works with a variety of identifiers including KEGG, INCHI, PUBMED, HMDB, CHEBI, LIPIDMAPS. This module is a flexible tool that allows both exact or class matching. By these means, 134 metabolites corresponding to 61% of the pool of identified metabolites in the samples were successfully mapped with exact matching on Recon3D. Unmapped metabolites may be missing from the network, but problems with identifier association may also occur. Over-representation analysis was carried out to gain mechanistic insight into altered metabolite subsets. This method highlights biological pathways that are enriched in a metabolite list more than would be expected by chance using a right-tailed Fisher test corrected using the Benjamini-Hochberg False Discovery Rate procedure. Subnetwork extraction was performed based on significantly over-represented metabolic pathways associated with the first NetPCA component.

Metabolic modules were finally evaluated based on their carbon transfer reaction path in using a distance matrix computed between the compounds of the selected subset of altered metabolites. Hierarchical clustering was then carried out in the Matlab environment to highlight potential biologically meaningful groupings based on the distance matrix. Complete-linkage was used as agglomerative hierarchical clustering method.

**References**

Brunk, E., S. Sahoo, D. C. Zielinski, A. Altunkaya, A. Drager, N. Mih, F. Gatto, A. Nilsson, G. A. P. Gonzalez, M. K. Aurich, A. Prlic, A. Sastry, A. D. Danielsdottir, A. Heinken, A. Noronha, P. W. Rose, S. K. Burley, R. M. T. Fleming, J. Nielsen, I. Thiele and B. O. Palsson (2018). "Recon3D enables a three-dimensional view of gene variation in human metabolism." Nature Biotechnology **36**(3): 272-+.

Chazalviel, M., C. Frainay, N. Poupin, F. Vinson, B. Merlet, Y. Gloaguen, L. Cottret and F. Jourdan (2018). "MetExploreViz: web component for interactive metabolic network visualization." Bioinformatics **34**(2): 312-313.

Codesido, S., M. Hanafi, Y. Gagnebin, V. González-Ruiz, S. Rudaz and J. Boccard (2020). "Network principal component analysis: a versatile tool for the investigation of multigroup and multiblock datasets." Bioinformatics.

Cottret, L., D. Wildridge, F. Vinson, M. P. Barrett, H. Charles, M. F. Sagot and F. Jourdan (2010). "MetExplore: a web server to link metabolomic experiments and genome-scale metabolic networks." Nucleic Acids Research **38**: W132-W137.

Pezzatti, J., V. Gonzalez-Ruiz, S. Codesido, Y. Gagnebin, A. Joshi, D. Guillarme, J. Schappler, D. Picard, J. Boccard and S. Rudaz (2019). "A scoring approach for multi-platform acquisition in metabolomics." Journal of Chromatography A **1592**: 47-54.
